# Follow-up of young patients after acute poisoning by substances of abuse: a comparative cohort study at an emergency outpatient clinic

**DOI:** 10.1186/s13104-016-2200-6

**Published:** 2016-08-09

**Authors:** Odd Martin Vallersnes, Mari A. Bjornaas, Cathrine Lund, Dag Jacobsen, Øivind Ekeberg, Mette Brekke

**Affiliations:** 1 Department of General Practice, University of Oslo, Oslo, Norway; 2 Oslo Accident and Emergency Outpatient Clinic, Department of Emergency General Practice, City of Oslo Health Agency, Oslo, Norway; 3 Department of Acute Medicine, Oslo University Hospital, Oslo, Norway; 4 Division of Mental Health and Addiction, Oslo University Hospital, Oslo, Norway; 5 Department of Behavioural Sciences in Medicine, University of Oslo, Oslo, Norway

**Keywords:** Acute poisoning, Substances of abuse, Brief intervention, Ethanol, Emergency medicine, Young patients, Adolescents, Follow-up

## Abstract

**Background:**

Young patients with acute poisoning by substances of abuse have increased mortality rates in the long term. In Oslo, Norway, most of these patients are treated at the Oslo Accident and Emergency Outpatient Clinic. The majority were discharged without follow-up. In 2010, the clinic implemented an intervention program for patients under the age of 23 presenting with acute poisoning by substances of abuse. The intervention was a brief motivational interview with a social worker before discharge, followed by a telephone consultation. Patients in need of further follow-up were identified and referred. Our objective was to study short-term effects of the intervention program on referrals to follow-up and repetition rates of acute poisoning.

**Methods:**

Comparative cohorts were derived from studies of acute poisoning at the Oslo Accident and Emergency Outpatient Clinic in 2003, 2008 and 2012. Two age groups of patients presenting with acute poisoning by substances of abuse were included: 16–22 years and 23–27 years. Patients in the pre-intervention cohorts of 2003 and 2008 were compared with patients of the same age in the post-intervention cohort of 2012. Repetition rates were estimated using survival analysis. In total, 1323 patients were included; 422 in the younger pre-intervention group, 366 in the younger post-intervention group, 288 in the older pre-intervention group, and 247 in the older post-intervention group. Overall, the major toxic agents were ethanol 823/1323 (62 %) and opioids 215/1323 (16 %). 719/1323 (54 %) patients were male.

**Results:**

In the younger groups referrals to follow-up increased from 86/317 (27 %) to 156/366 (43 %) (p < 0.001) after the implementation of the program. Among the older patients, who were not included in the program, there was no significant change in referrals. There was no change in the repetition rate of acute poisoning in either age group. The program established contact with 225/366 (61 %) of the eligible patients.

**Conclusion:**

More patients were referred to follow-up after the intervention. We expect this to have a beneficial effect on their substance use and reduce excess morbidity and mortality in the long term. There was no change in the repetition rate of poisoning.

## Background

Acute poisoning is mainly due to suicidal behaviour or the result of substance abuse. Mortality rates are increased for this patient group in the long term [1], and the excess mortality is due to both natural and unnatural causes [1, 2]. Young patients and substance abusers are especially at risk [1–5]. An acute poisoning episode is not only a marker of increased risk, but also an opportunity for intervention [6–8]. Brief motivational interventions are found to be effective in reducing harmful and hazardous drinking [9, 10]. Emergency department brief interventions for young patients using alcohol or other substances of abuse seem promising, but systematic reviews remain inconclusive [11–13].

In Oslo, Norway, the majority of patients with acute poisoning are treated at an emergency outpatient clinic, twice as many as at the city’s hospitals combined [14]. About 80 % of the acute poisonings treated at the emergency outpatient clinic are caused by substances of abuse, including ethanol and benzodiazepines [14, 15]. In 2008, 53 % of the poisoned patients were discharged without follow-up [14]. Patients with substance abuse related poisoning are less likely to be referred compared to patients whose poisoning was a suicide attempt, despite higher mortality rates [1, 14–16]. Hence, in 2010, an intervention program for patients under the age of 23 presenting with acute poisoning by substances of abuse was established at the emergency outpatient clinic. The age limit was set at 23 years, as patients below this age by Norwegian law have a higher priority for treatment in the specialist health service for substance abuse and addiction [17, 18]. Patients in the target age were eligible for the intervention irrespective of the intention behind the poisoning.

## Objectives

Our main aim was to study the effect of the intervention program for young patients treated for acute poisoning by substances of abuse, by comparing referrals to follow-up and repetition rates of acute poisoning before and after the implementation of the program in 2010. We also wanted to identify factors associated with repetition, from the available data.

## Methods

**Fig. 1:**
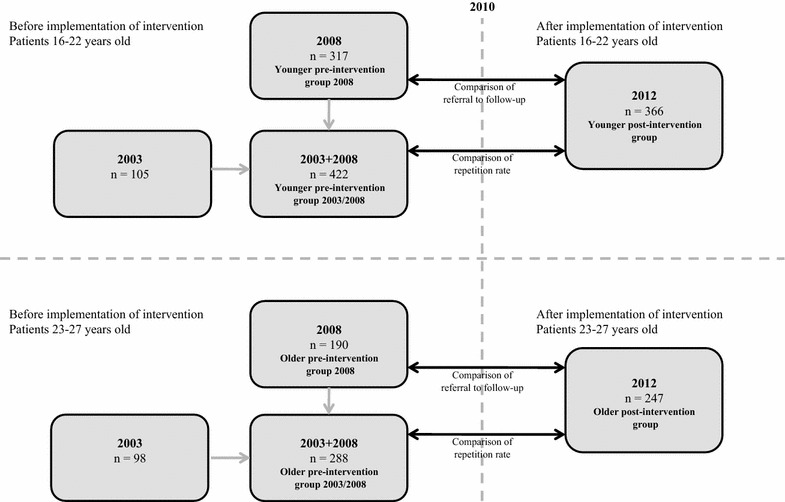
Cohorts and comparisons. The cohorts were made from three separate studies of acute poisoning at the Oslo Accident and Emergency Outpatient Clinic (OAEOC) in 2003, 2008, and 2012. The intervention program for patients under the age of 23 years presenting with acute poisoning by substances of abuse was implemented at the OAEOC in 2010. Repetition rates and proportions of patients referred to follow-up were compared before and after the implementation. Comparisons were made for patients in the target age, and for patients just older than the targeted age group. Referral to follow-up was not registered in the 2003 study

The study was a comparative cohort study, comparing a cohort exposed to the intervention with cohorts not exposed to the intervention (Fig. 1). The study was part of a larger prospective observational study of acute poisoning conducted at the Oslo Accident and Emergency Outpatient Clinic (OAEOC) in 2012 [19]. The cohorts were derived from this study and from similar studies done in 2003 [15] and 2008 [14]. All three studies registered all acute poisonings at the OAEOC during one year. The study periods were April 1st 2003 to March 31st 2004, April 15th 2008 to April 14th 2009, and October 1st 2011 to September 30th 2012. Patient inclusion criteria were identical in all the three study periods.

### Setting

In Norway, patients cannot present directly to hospitals, but have to be assessed in primary care or by the ambulance service first. Primary care emergency services are provided by regular general practitioners during office hours, and by local casualty clinics during nights and weekends. The OAEOC is the main casualty clinic in Oslo. It serves the entire city (population 613,285 as per January 1st 2012 [20]) at all hours. There are facilities for short time observation, but diagnostic tools and treatment options are limited. The total number of consultations at the OAEOC was about 180,000 in 2003, and about 200,000 in 2008 and 2012. In 2012, about 3000 consultations were due to acute poisoning [19].

### Participants

Patients 16–27 years of age presenting at the OAEOC during the study periods with an acute poisoning in which the main agent was a substance of abuse, were included. Substances of abuse were defined as any drug used for recreational purposes or with a known potential for addiction or abuse, including ethanol, prescription drugs and other substances. Patients with uncertain identity, i.e. without a Norwegian national identity number, were not included. The patients were divided into four groups based on being in the target age for the intervention (16–22 years) or not (23–27 years), and on being included prior to or after the implementation of the intervention. The patients eligible for the intervention were thus the younger post-intervention group (Fig. 1). The older groups (23–27 years) were included as comparisons. The rationale was that changes in referrals or repetition rates across the target age groups were likely also to show up in the older groups if due to other factors than the intervention. Hence, we decided to use groups of patients just older than the target age groups (16–22 years). The upper limit of 27 years was a compromise between being high enough to include an adequate number of patients and low enough not to include patients too different in sociodemographic characteristics due to being older.

In total, 1323 patients were included. The major toxic agents at the index episode were ethanol 823 (62 %) and opioids 215 (16 %). 719 (54 %) patients were male. Nearly all the poisonings were accidental overdoses (Table 1).

**Table 1:** Demographic data

	Age 16–22 years		Age 23–27 years	
	2012 younger post-intervention n (%)	2008 + 2003 younger pre-intervention n (%)	2012 older post-intervention n (%)	2008 + 2003 older pre-intervention n (%)
*Males*	188 (51)	207 (47)	151 (61)	173 (60)
*Oslo residents*	188 (51)*	195 (62)^a^	138 (56)	114 (60)^b^
*Hospitalised*	39 (11)	56 (13)	38 (15)	40 (14)
*Main toxic agents*				
Ethanol	271 (74)	292 (69)	127 (51)	133 (46)
Opioids	30 (8)*	55 (13)	47 (19)*	83 (29)
Benzodiazepines	25 (7)	38 (9)	23 (9)	22 (8)
Central stimulants	13 (4)	11 (3)	12 (5)	26 (9)
GHB^c^	14 (4)	20 (5)	20 (8)	16 (6)
Other	13 (4)	6 (1)	18 (7)*	8 (3)
*Intention*				
AOSA^d^	345 (94)	286 (90)^a^	222 (90)	166 (87)^b^
Suicidal	16 (4)	20 (6)^a^	13 (5)	18 (9)^b^
Other	5 (1)	11 (3)^a^	12 (5)	6 (3)^b^
*Total*	366 (100)	422 (100)	247 (100)	288 (100)

Demographic data for 1323 patients aged 16–27 years treated for acute poisoning by substances of abuse at the Oslo Accident and Emergency Outpatient Clinic in 2003, 2008 and 2012

The intervention program for patients under the age of 23 was established in 2010

p-values are for comparisons of frequencies before and after the implementation of the intervention program, using Pearson’s Chi square test or Fisher’s exact test (for expected cell counts of five or less)

* p < 0.05. No p-values were lower than 0.01

^a^Not registered in 2003 study, thus patients from 2008 cohort only, n = 317, percentage calculated accordingly

^b^Not registered in 2003 study, thus patients from 2008 cohort only, n = 190, percentage calculated accordingly

^c^Gamma-hydroxybutyrate

^d^Accidental overdose with substances of abuse

### Intervention

The intervention program consisted of consultations with specially trained social workers. Patients in the target age treated for acute poisoning by substances of abuse were contacted by a social worker for a consultation before discharge. An appointment was also made for a telephone consultation within two weeks, unless the patient declined or the social worker deemed it unnecessary. Patients not contacted before discharge, were contacted by telephone within a week and offered similar consultations. Letters were sent to patients not reached by telephone, encouraging them to contact the intervention program. Parents were contacted if the patient was a minor (<18 years). In the consultations, both face to face and by telephone, the social workers used motivational interviewing [21, 22]. The main aim was to reduce the hazardous use of substances of abuse. In addition, AUDIT (Alcohol Use Disorders Identification Test) [23] and DUDIT (Drug Use Disorders Identification Test) [24] were used to identify patients in need of further follow-up, and to ensure that these patients were referred or already in relevant treatment. Over and above these elements, the consultation content was left to the discretion of the social worker and tailored to the individual patient.

### Outcome measures

We compared proportions of patients referred to follow-up and repetition rates of poisoning. The comparisons were intention-to-treat analyses. Thus, the younger post-intervention group encompassed all patients eligible for the intervention, whether or not they were in contact with the program.

Referral to follow-up was defined as hospital admission, addiction clinic admission, referral to a psychiatric outpatient clinic, the patient’s general practitioner, child welfare services, social services, or ensuring that the patient already was in such treatment. Discharge with no follow-up or the patient self-discharging was also registered. When comparing proportions of patients referred to follow-up, patients from the 2003 cohort were not included, as referrals were not registered in this study period (Fig. 1).

The poisoning episode resulting in inclusion was considered the index episode. Subsequent episodes for the same patient in the same study period were considered repetition episodes.

Contact with the intervention program was defined as at least one consultation with program personnel, face to face or by telephone. A voicemail or just a short conversation was not considered a consultation.

### Data collection

The poisoning episodes were recorded consecutively. The treating physician registered the following data on a pre-set registration form: date, age, sex, residence (2008 and 2012 studies only), main toxic agent, intention behind the poisoning (2008 and 2012 studies only), admission to hospital and referral to treatment or follow-up (2008 and 2012 studies only). We examined electronic patient records for patients in the younger post-intervention group, and registered all contacts with the program. Data on death and emigration were retrieved from the National Registry. Norwegian national identity numbers, unique for every inhabitant, were used to identify patients.

Toxic agents (ethanol, opioids, benzodiazepines, central stimulants, gamma-hydroxybutyrate (GHB), or other) were diagnosed by the physician treating the patient, based on all information available at the time: patient history; information from the police, the ambulance service, relatives or other companions; and clinical examination. No toxicological tests were available, apart from breath analysis for ethanol. Intention was clinically assessed by the treating physician, and registered as suicidal intention, accidental overdose by substances of abuse, mere accident, or unknown.

### Ethics

The study was performed in accordance with the Helsinki declaration. Inclusion was based on verbal consent. The study was approved by the Regional Committee South East for Medical and Health Research Ethics (REK nr 2010/1129-1) and by the Oslo University Hospital Information Security and Privacy Office.

### Statistics

Analyses were done in IBM SPSS version 21 (IBM Corp.). Pearson’s Chi square test or Fisher’s exact test (for expected cell counts of five or less) were used to compare frequencies.

Repetition rates were estimated by survival analysis. An event was defined as the first repetition episode. Time under observation was from the index episode to the event, or until censored by death, emigration, or the end of the relevant study period, whichever happened first. Time under observation was counted in number of days, as integers. If the first repetition occurred on the same day as the index episode, the time under observation was set to one day.

Cox regression analysis was used to estimate hazard ratios for factors associated with repetition of poisoning, with all cohorts pooled together. In the Cox regression analysis relevant variables were first analysed one by one. The variables analysed were age, sex, main toxic agent, hospitalisation, suicidal intention and self-discharge from the OAEOC. Ethanol was chosen as the reference group when estimating hazard ratios for main toxic agents, as it was the largest group. Factors associated with repetition in the univariate analyses with a significance level of p < 0.10 were included in the multivariate model. We did a separate Cox regression analysis to look for association between hazard of repetition and referral to follow-up. We used the same model, but only on the 2008 and 2012 cohorts, as referral to follow-up was not registered in 2003. We did not include hospitalisation in this analysis, as hospitalisation was categorised as being referred to follow-up.

## Results

### Comparison of referrals to follow-up

In the younger pre-intervention group 86/317 (27 %) patients were in or referred to follow-up, whereas in the younger post-intervention group there were 156/366 (43 %), an increase of 57 % (relative risk 1.57, 95 % CI 1.27–1.95, p < 0.001). In the older pre-intervention group 68/190 (36 %) patients were in or referred to follow-up, and in the older post-intervention group there were 101/247 (41 %), an increase of 14 % (relative risk 1.14, 95 % CI 0.90–1.46, p = 0.28).

Table 2 shows follow-up before and after the implementation of the program. More patients in the younger post-intervention group were referred to psychiatric outpatient clinics, 57/366 (16 %), versus 13/317 (4 %), (p < 0.001) in the younger pre-intervention group.

**Table 2:** Referral to follow-up

	16–22 years		23–27 years	
	2012 younger post-intervention n (%)	2008 younger pre-intervention n (%)	2012 older post-intervention n (%)	2008 older pre-intervention n (%)
Admitted hospital psychiatric	6 (2)	8 (3)	4 (2)	6 (3)
Admitted hospital somatic	33 (9)	34 (11)	34 (14)	24 (13)
Addiction clinic	3 (1)	0 (0)	12 (5)**	0 (0)
Psychiatric outpatient clinic	57 (16)***	13 (4)	21 (9)	13 (7)
Child welfare services	19 (5)**	3 (1)	0 (0)	0 (0)
Social services	20 (5)	11 (3)	14 (6)	10 (5)
General practitioner	16 (4)	8 (3)	6 (2)	8 (4)
Other	2 (1)*	9 (3)	10 (4)	7 (4)
Discharged, no follow-up	195 (53)*	197 (62)	118 (48)	94 (49)
Self-discharged	15 (4)**^a^	34 (11)	28 (11)	28 (15)
Total referred to follow-up	156 (43)***	86 (27)	101 (41)	68 (36)
*Total*	366 (100)	317 (100)	247 (100)	190 (100)

Referral to follow-up after acute poisoning by substances of abuse before and after the implementation of the intervention program in 2010

Highest level of admission or referral initiated at index poisoning episode, or later referral by the program

p-values are for comparisons of frequencies before and after the implementation of the intervention program, using Pearson’s Chi square test or Fisher’s exact test (for expected cell counts of five or less)

* p < 0.05, ** p < 0.01, *** p < 0.001

^a^At the index episode 25 (7 %) patients self-discharged. Ten of them were later contacted by the intervention program and referred to follow-up

### Comparison of repetition rates

**Fig. 2:**
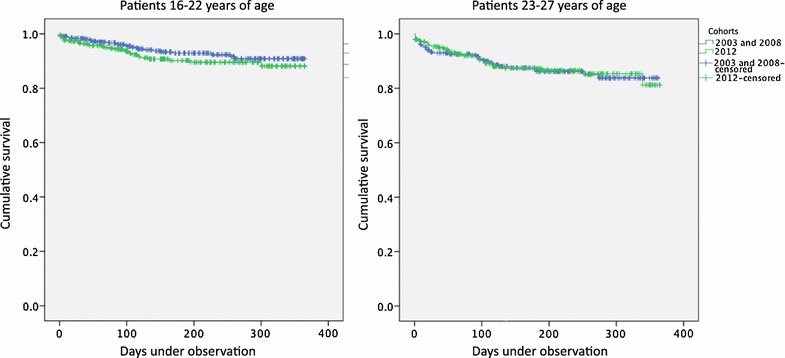
Repetition rates. Repetition rates of acute poisoning by substances of abuse at the Oslo Accident and Emergency Outpatient Clinic (OAEOC). Kaplan–Meier plots of repeated poisoning before and after implementation of the intervention program, patients in target age (*left panel*), and patients just older than target age (*right panel*). *Left panel* younger pre-intervention group (*blue*; n = 422, 27 events, 393 censored at end of study, two censored due to emigration), and younger post-intervention group (*green*; n = 366, 31 events, 335 censored at end of study). No significant difference between groups, log rank test (Mantel–Cox) gives Χ^2^ = 1.597 (p = 0.21). Cumulative repetition probability (95 % CI) in younger pre-intervention group estimated at 9 % (6–13 %), and in younger post-intervention group at 12 % (7–16 %). When the last repetition occurred, 62 patients were still under observation. Time under observation ranged from 1 to 365 days in both groups.* Right panel* Older pre-intervention group (*blue*; n = 288, 36 events, 252 censored at end of study), and older post-intervention group (*green*; n = 247, 29 events, 217 censored at end of study, one censored due to death). No significant difference between groups, log rank test (Mantel–Cox) gives Χ^2^ = 0.0014 (p = 0.91). Cumulative repetition probability (95 % CI) in older pre-intervention group estimated at 16 % (11–22 %), and in older post-intervention group at 19 % (10–28 %). When the last repetition occurred, 20 patients were still under observation. Time under observation ranged from 1 to 362 days in older pre-intervention group, and from 1 to 365 days in older post-intervention group

There was no significant difference in the repetition rate between the younger groups, as shown in Fig. 2. Cumulative repetition probability in the younger pre-intervention group was estimated at 9 % (95 % CI 6–13 %) and in the younger post-intervention group at 12 % (95 % CI 7–16 %). There was no significant difference in the repetition rate between the older groups, either. Cumulative repetition probability in the older pre-intervention group was estimated at 16 % (95 % CI 11–22 %) and in the older post-intervention group at 19 % (95 % CI 10–28 %).

Some patients had more than one repetition episode during the study period; 6/422 (1 %) in the younger pre-intervention group, 10/366 (3 %) in the younger post-intervention group, 15/288 (5 %) in the older pre-intervention group, and 9/247 (4 %) in the older post-intervention group. The maximum number of repetitions was eight.

### Factors associated with repetition

Patients presenting with opioid poisoning had a nine times higher adjusted hazard (95 % CI 6–14, p < 0.001) of repeated poisoning compared to patients with ethanol poisoning (Table 3). The adjusted hazard of repeated poisoning was also higher for patients presenting with poisoning with benzodiazepines, central stimulants, and GHB. No association was found between hazard of repeating and age, sex, hospitalisation, suicidal intention or self-discharge. Neither was there any association between repetition hazard and referral to follow-up (Table 4).

**Table 3:** Factors associated with repeated poisoning—Cox regression analysis

			Crude			Adjusted		
	n	Events	Hazard ratio	95 % CI	p	Hazard ratio	95 % CI	p
*Age*								
16–22 years^a^	788	58						
23–27 years	535	65	1.7	1.2–2.4	0.004	1.1	0.78–1.6	0.525
*Sex*								
Females^a^	604	45						
Males	719	78	1.5	1.0–2.2	0.033	1.1	0.77–1.6	0.553
*Main toxic agent*								
Ethanol^a^	823	30						
Opioids	215	59	9.3	6.0–14.4	<0.001	8.9	5.6–14.1	<0.001
Benzodiazepines	108	16	4.7	2.5–8.6	<0.001	4.8	2.5–8.9	<0.001
Central stimulants	62	6	2.8	1.2–6.7	0.022	2.6	1.1–6.4	0.032
GHB	70	9	3.6	1.7–7.7	0.001	3.7	1.7–8.3	0.001
Other	45	3	1.9	0.58–6.2	0.288	1.8	0.56–6.1	0.316
*Outcome*								
Not hospitalised^a^	1150	100						
Hospitalised	173	23	1.6	1.0–2.5	0.050	0.91	0.56–1.5	0.706

Factors associated with repetition of poisoning in 1323 patients aged 16–27 years presenting with acute poisoning by substances of abuse in 2003, 2008 and 2012. There were 123 events (repetitions). One patient was censored due to death, two were censored due to emigration

^a^Reference group

**Table 4:** Referral and hazard of repeated poisoning—Cox regression analysis

	n	Events	Crude			Adjusted		
			Hazard ratio	95 % CI	p	Hazard ratio	95 % CI	p
*Age*								
16–22 years^a^	683	50						
23–27 years	437	54	1.7	1.2–2.5	0.005	1.1	0.74–1.7	0.62
*Sex*								
Females^a^	518	37						
Males	602	67	1.6	1.1–2.4	0.021	1.2	0.76–1.8	0.50
*Main toxic agent*								
Ethanol^a^	715	27						
Opioids	170	48	9.2	5.7–14.8	<0.001	8.5	5.0–14.3	<0.001
Benzodiazepines	89	12	3.9	2.0–7.7	<0.001	3.8	1.8–7.8	<0.001
Central stimulants	49	6	3.5	1.4–8.5	0.006	3.2	1.3–8.0	0.012
GHB	60	8	3.7	1.7–8.1	0.001	3.4	1.5–7.9	0.004
Other	37	3	2.2	0.66 -7.1	0.20	2.1	0.63–6.9	0.23
*Referral to follow*-*up*								
No referral^a^	709	55						
Referral	411	49	2.0	1.4–2.9	<0.001	1.0	0.69–1.6	0.83

Referral to follow-up and hazard of repetition of poisoning in 1120 patients aged 16–27 years presenting with acute poisoning by substances of abuse in 2008 and 2012

There were 104 events (repetitions). One patient was censored due to death, two were censored due to emigration

The hazard ratio for referrals was hardly affected by entering age and sex into the model, but changed when main toxic agent was entered

^a^Reference group

### Contact with the intervention program

The program established contact with 225 (61 %) of the 366 eligible patients. Among them, 99 (44 %) had consultations both before discharge and later by telephone, 72 (32 %) had a telephone consultation only, 51 (23 %) had a consultation before discharge only, and three (1 %) had a first face-to-face consultation at a later time. Median time from poisoning episode to telephone consultation was seven days (range 0–89 days, interquartile range 1–15 days). Nine (4 %) patients had more than one telephone consultation. Ten (4 %) had a second face-to-face consultation with the program. Table 5 shows differences between the patients who were in contact with the program and those who were not.

**Table 5:** Patients in and not in contact with the intervention program

	In contact n (%)	Not in contact n (%)
*Males*	116 (52)	72 (51)
*Oslo residents*	120 (53)	68 (48)
*Presented in weekend*	154 (68)***	57 (40)
*Suicidal intent*	5 (2)*	11 (8)
*Main toxic agents*		
Ethanol	190 (84)***	81 (57)
Opioids	11 (5)**	19 (13)
Benzodiazepines	7 (3)**	18 (13)
Central stimulants	7 (3)	6 (4)
GHB^a^	3 (1)**	11 (8)
Other	7 (3)	6 (4)
*Follow*-*up* ^b^		
Admitted psychiatric hospital	2 (1)	4 (3)
Admitted somatic hospital	13 (6)*	20 (14)
Addiction clinic	2 (1)	1 (1)
Psychiatric outpatient clinic	47 (21)	23 (16)
Child welfare services	15 (7)	8 (6)
Social services	23 (10)*	28 (20)
General practitioner	24 (11)*	6 (4)
Contact with parents	23 (10)**	3 (2)
*Total*	225 (100)	141 (100)

Differences between targeted patients in and not in contact with the intervention program for young patients with acute poisoning by substances of abuse in 2012. The program established contact with 225 (61 %) of the 366 eligible patients

^a^Gamma-hydroxybutyrate

^b^Some patients were referred to and/or already in treatment at several services

* p < 0.05

** p < 0.01

*** p < 0.001

Among the 141 patients not in contact with the program, 67 (48 %) were already in or referred to follow-up, leaving 74/366 (20 %) of the eligible patients not in contact with the program, and not in or referred to follow-up. Among them, 36/74 (49 %) were males, 8/74 (11 %) self-discharged, and the main toxic agent was ethanol in 64/74 (86 %). Letters were sent to 51 of these 74 patients. The main reason for not sending letters was lacking contact information.

Five patients 23 years of age, thus in the older post-intervention group, were in contact with the program despite being too old.

## Discussion

### Summary of main results

The proportion in or referred to follow-up increased significantly among patients in the target age after the implementation of the program, from 86/317 (27 %) in 2008 to 156/366 (43 %) in 2012. There was no significant change in the repetition rate of acute poisoning. Patients treated for opioid poisoning had the highest risk of repetition.

## Limitations

Ideally, we should have done a randomised controlled trial. However, this study was part of a larger study of acute poisoning at the OAEOC in 2012. At the time of planning this study, the intervention program was already up and running. Though similar interventions have not conclusively been shown to be effective [11, 12], the intervention program had been implemented on the presumption of being beneficial. On this background, we considered it an ethical problem to deprive randomly selected patients of an offer they were entitled to. Hence, we decided against doing a randomised controlled trial and chose to do a comparative cohort study instead.

We consider the cohorts comparable despite them being recruited at different times. The patients were included in their respective cohorts in the same way, using the same criteria. The cohorts were fairly similar on the studied parameters (Table 1). There were no major changes in local treatment protocols at the OAEOC or in ambulance triage procedures between the studies.

There was probably some variation in how the intervention was done, as several social workers were involved, and the guidelines were not strict. Much was left to the individual social worker’s judgment in each particular consultation. Such variations are to be expected in a study done in a real clinical setting. Then again, real clinical settings are where such interventions are implemented. We do not know whether referred patients actually ever presented at the clinics they were referred to. It is likely that some did not [25]. The number of patients already in treatment is based on information from the patients themselves. It is likely that some patients were not asked. Patients in contact with the program were possibly asked more frequently.

Diagnosis of toxic agents and assessment of intention was based on clinical examination and all information available then and there. This limits the accuracy of the diagnoses, but mirrors the actual clinical situation these patients are treated in. There may be considerable inter-rater variability as about 70 different physicians are employed at the Department of Emergency General Practice of the OAEOC at any one time, but most likely equally common in all groups.

### Referrals to follow-up

A significantly larger proportion of patients in the target age group was in or referred to follow-up after the implementation of the intervention program. An acute poisoning by substances of abuse can be seen as a marker for at-risk alcohol or substance use [1, 3, 16, 26]. Getting more of these patients in treatment or other relevant follow-up is an important achievement as patients in treatment reduce their alcohol and substance use, and hence their risk of death [9, 22, 27, 28].

No association was found between hazard of repetition and being in or referred to follow-up. Thus, referral would not seem to reduce the risk of repeated poisoning. On the other hand, it is probable that the patients referred were those with more at-risk substance use and mental health problems, at the same time being the ones most prone to repeat.

### Repetition rates

No change was found in one-year repetition rates after the implementation of the program. The time frame was probably adequate, as the risk of repetition is highest during the first year [29, 30]. However, the number of patients in the study was not large enough to detect small but possibly clinically significant differences. The wide confidence intervals of the repetition rate estimates (Fig. 2) suggest that changes of up to about five percentage points would go undetected among the younger patients, as would changes of up to about ten percentage points among the older patients, given the number of patients included in the study.

The repetition rate in this study was estimated solely on the basis of poisonings treated at the OAEOC. Though the majority of poisonings with substances of abuse in Oslo are treated at the OAEOC, the more severe ones are brought directly to hospital by the ambulance service [15, 31]. A substantial number are also left on site after treatment by the ambulance service [15]. Nearly half the patients were not permanent Oslo residents, and may have been treated elsewhere for repeated poisoning. Most studies have shown repetition rates of about 15 % [30], but a study of repeated poisoning in Oslo in 2003, encompassing all levels of health care, found a one-year repetition rate of 30 %, though lower among young patients [32]. The real repetition rate among the patients is probably somewhat higher than estimated by us. Still, we consider our repetition rates a reliable measure for comparison across the groups, with the limitations previously discussed, as the basis for estimating the repetition rate was the same in all the three cohorts in the study.

### Factors associated with repetition

Main toxic agent was the only factor associated with increased hazard of repetition in this study. The hazard was especially increased for opioids, in consistence with other studies [29, 32]. There were fewer patients with opioid poisoning in 2012 than in the pre-intervention cohorts (Table 1), possibly due to the expansion of opioid maintenance therapy programs [33]. However, the overall repetition rates did not decrease. It is possible that the group of patients with heroin poisoning has changed as the opioid maintenance therapy programs have expanded. They may have become a more troubled group of patients, not managing to enter or remain in opioid maintenance programs, hence with a higher risk of repetition [34].

The factors analysed were limited by the collected data set. We did not collect any data on socioeconomic status or severity of alcohol or substance use, differences in which may also have contributed to the differences in the hazard ratios.

### Contact with the intervention program

The OAEOC was better staffed with social workers during weekend nights, when most young poisoned patients present, improving the chances of establishing contact while the patient was at the clinic. Most poisoned patients presenting during weekend nights have ethanol poisonings. Patients with opioid or benzodiazepine poisoning present all week, and were less likely to get in contact with the program. It is also possible that these patients were more difficult to establish contact with, being more troubled patients, in terms of having more complex health problems, being homeless and having weaker links to health care and other institutions. Patients admitted to hospital were less likely to be contacted by the program, as it was assumed that follow-up for these patients was taken care of by the hospital. Most patients with GHB poisoning were according to OAEOC procedure admitted to hospital. Hence, few were contacted by the program.

## Conclusion

After the implementation of a brief intervention program delivered by social workers, more young patients treated for acute poisoning by substances of abuse were referred to follow-up. We expect this will have a beneficial effect on their substance use and excess morbidity and mortality in the long term, although no immediate effect was seen on repetition rates. Patients with opioid poisoning had the highest hazard of repetition, highlighting that this group is at special risk.
